# Therapeutic Relationship and Dropout in High-Risk Adolescents’ Intensive Group Psychotherapeutic Programme

**DOI:** 10.3389/fpsyg.2020.533903

**Published:** 2020-11-27

**Authors:** Kirsten Hauber, Albert Boon, Robert Vermeiren

**Affiliations:** ^1^Curium-LUMC, Oegstgeest, Netherlands; ^2^Youz, The Hague, Netherlands

**Keywords:** dropout, therapeutic relationship, residential mental health care, adolescents, MBT

## Abstract

**Objective:**

Dropout rates are a prominent problem in youth psychotherapy. An important determinant of dropouts is the quality of the therapeutic relationship. This study aimed to evaluate the association between the therapeutic relationship and dropouts in an intensive mentalization-based treatment (MBT) for adolescents with personality disorders.

**Methods:**

Patients (*N* = 105) included were either dropouts (*N* = 36) or completers (*N* = 69) of an intensive MBT. The therapeutic relationship was measured with the child version of the Session Rating Scale (C-SRS), which was completed by the patient after each group therapy session. For each patient, the treatment termination status (dropout or completer) was indicated by the treatment staff. The reliable change index (RCI) was calculated for the C-SRS to determine significant changes in the therapeutic relationship.

**Results:**

While both groups started with similar scores on the C-SRS, the scores between dropouts and completers differed significantly at the end of the treatment period. On average, during therapy, an increase was seen in the scores of completers, and a decrease was seen in the scores of dropouts. While dropouts could not be predicted based on the C-SRS scores, a significant decrease (RCI) in C-SRS scores during the last two sessions occurred more often for dropouts than for completers.

**Conclusion:**

Our findings show that to prevent dropouts, the patient’s judgment of the quality of the therapeutic relationship should be monitored continuously, and decreases discussed with the patient and the group.

## Introduction

Dropout is a common phenomenon in child and adolescent therapy (De Haan et al., 2013; Owen et al., 2016; Hauber et al., 2017). When youngsters drop out of psychiatric treatment, their disorders are thought to persist or even worsen later in life. For instance, children with untreated disorders are more likely to become adults who rely on mental health services, having negative consequences for themselves, their surroundings, and society (Dulmus and Wodarski, 1996; Reis and Brown, 1999; Kessler et al., 2005). Premature termination of therapy is therefore considered a serious problem (Armbruster and Kazdin, 1994; Midgley and Navridi, 2006; Gopalan et al., 2010). Dropout percentages of 38.4% for outpatients (De Haan et al., 2015) and 34.4% for inpatients (De Boer et al., 2016) were found in adolescent mental health care. An important determinant for dropout is the quality of the therapeutic (patient–therapist) relationship (Kazdin and Wassell, 1998; Garcia and Weisz, 2002; Hawley and Weisz, 2005; Stevens et al., 2006; De Haan et al., 2013; Owen et al., 2016). For this reason, this study aimed to evaluate the association between the therapeutic relationship and dropout in an intensive mentalization-based treatment (MBT) for adolescents with personality disorders.

A therapeutic relationship or therapeutic alliance has commonly been defined as an agreement between the therapist and client on the goals for treatment, as well as ways to reach those goals, and the emotional or relational bond between the client and therapist (Bordin, 1979). Although several studies have been conducted on the relation between therapeutic relationship and dropout, it is hard to compare these studies because the time at which the therapeutic relationship was measured varies considerably (Robbins et al., 2003, 2006; Shelef et al., 2005; Cordaro et al., 2012). In several studies, trained observers rated therapeutic alliance based on one or two therapy sessions during the course of therapy. This approach, however, does not take the patients’ opinion about the relationship into account. Other studies measured the relationship from the patients’ point of view, but only after therapy had ended. These measurements would be strongly influenced by the way the patients felt at the time of termination. In a review on the therapeutic relationship within youth therapy, it is advised to measure the therapeutic relationship from the patients’ point of view during several sessions of the therapy process (Zack et al., 2007). If adolescents perceive the therapeutic relationship as supportive and agree with the topics and goals of the sessions, it will facilitate their engagement (Karver et al., 2006). One study, examining both the adolescents’ and the therapists’ perspectives on the therapeutic alliance, found that the client provided more information than the therapist did (Ormhaug et al., 2015). However, to this day, adolescent patients are hardly used as informants about the therapeutic alliance (De Haan et al., 2013).

In adult therapy, a moderately strong relationship between psychotherapy dropout and therapeutic alliance is found (Sharf et al., 2010). In youth therapy, studies on the relationship between the therapeutic alliance and dropout have been hindered in two ways: (1) the methods in which the therapeutic relationship could be measured and (2) the definition of dropout. First, most available measures for the therapeutic relationship in child and adolescent therapy are parent-report measures. The Therapeutic Alliance Scale for Children and Adolescents (TASC/A) is an exception and was designed to be administered to children and adolescents themselves (Shirk and Saiz, 1992; DeVet et al., 2003; Kazdin et al., 2005). The TASC/A was however designed to only be administered at one or two sessions throughout the course of therapy. The only available child-report instrument that measures the therapeutic relationship during all sessions is the child version of the Session Rating Scale (C-SRS) (Duncan et al., 2003, 2006; Miller and Duncan, 2004). This instrument is designed to be used at the end of every therapy session, and the child version of this tool makes it possible to assess the child’s or adolescent’s self-reported relationship with the therapist. Although designed for individual therapy, the instrument can also be used for group therapy. When used for group therapy, the therapeutic relationship is defined with three interacting elements: (1) a relational bond between the therapists, the group members, and the patient; (2) agreement on the goals of therapy; and (3) agreement on the tasks of therapy. The second topic that complicates research in this field is that there is also no agreement in the way dropout is defined. The definition varies across studies and influences which dropout predictors were found per study (De Haan et al., 2013, 2014a; Zack et al., 2007; Warnick et al., 2012). In this study, therapy dropout was defined as occurring when a participant discontinued the treatment programme before completing the treatment protocol, meaning that participants completing the treatment programme as planned were considered completers.

The aim of our study was to extend and specify insights on the association between the therapeutic relationship from the patients’ point of view and dropout during adolescent group psychotherapy. In accordance with Zack et al. (2007), we measured the therapeutic alliance of each psychotherapy session with an authorized Dutch version of C-SRS (Duncan et al., 2006; Hafkenscheid et al., 2006). Studies evaluating the (C-)SRS have confirmed the psychometric quality and usability of the instrument and have shown an association between therapeutic relationship and therapeutic change or outcome (Duncan et al., 2003; Campbell and Hemsley, 2009; Boon et al., 2012; Sundet, 2012; Owen et al., 2016). The association between the C-SRS and dropout has been studied in a sample of ethnic minority youth (De Haan et al., 2014a). Moreover, it was also shown that the scores on the C-SRS were not influenced by the patient’s knowledge of whether the scores would be observed by the therapists or not or whether the questionnaires were completed in the presence of the therapists. Moreover, the (C-)SRS scores were not significantly correlated with a measure of social desirability (Reese et al., 2013).

## Materials and Methods

### Setting

The studied facility, a department of De Jutters-Youz, a YMHC center in The Hague (one of the three most important cities of the Netherlands), offers a 5 day/week structured and integrative psychodynamic group psychotherapy programme. The aforementioned treatment commonly starts as a residential treatment and becomes an outpatient treatment halfway through. It is an MBT programme, manualized and adapted for adolescents (Bateman and Fonagy, 2006, 2012; Hauber, 2010), facilitated by a multidisciplinary team trained in MBT. The programme differs from the MBT programme for adolescents in England (Rossouw and Fonagy, 2012) in the psychodynamic group psychotherapy approach with an optimal group therapy size of six members instead of eight. The different components of the treatment programme mainly focus on the adolescents’ subjective experience of himself or herself and others and on the relationships with group members and treatment staff. Alongside the weekly group psychotherapy conducted by two therapists, other (non-verbal) group therapies such as art therapy and psychodrama therapy, as well as individual and family psychotherapy, are offered. In case medication is needed in addition to the treatment, this is prescribed by a psychiatrist in the team according to protocol. Weekends are spent at home.

In the weekly open group psychotherapy, as in the other components of the treatment programme, the therapists’ goal is to establish the group as a training ground for mentalizing. During the 1.5 h-long group therapy, all group members are invited to share their problems and to focus not only on what is shared but also on how things are shared by each group member. This often causes the therapeutic alliance to be a topic of conversation. Sometimes, one of the two group psychotherapists was also the individual therapist or EMDR therapist of one of the group members. In 2013, hoping to reduce dropout, the staff decided to measure the therapeutic relationship from the adolescents’ point of view and use it as input for the treatment offered. To this end, the C-SRS was administrated at the end of every weekly group psychotherapy. No sample size for power analysis was calculated beforehand, but the data of a 5-year period were used for this study.

### Participants

The participants were a sample of 105 patients with clinically diagnosed personality disorders according to the DSM-III (APA, 2013) admitted between 2013 and 2018. Co-morbid pervasive developmental disorder and psychosis was set as an exclusion criterion. Intelligence was not measured but, based on the level of education, was estimated to be average to above average. Most patients (94.4%) had a native Dutch background, and the Dutch language was fluently spoken by all participants.

Upon arrival, patients and their parents were asked to sign a consent form to indicate that their data could be used anonymously for scientific research. At the start of the treatment, the mean age of the adolescents was 17.7 (*SD* = 1.7, range = 15–22) (females 81.0%). Average duration of treatment during this study was 215.2 days (*SD* = 100.8, range 21–640), for completers (*M* = 261.9, *SD* = 63.2, range = 168–640) and dropouts (*M* = 125.6, *SD* = 99.1, range = 21–343). Most of the patients (90.4%) were clinically diagnosed with a personality disorder often with co-morbid axis I disorders (mood disorder 48.5%, anxiety disorder including PTSS 57.3%, eating disorder 8.7%, ADHD 7.6%, substance dependence 3.9%, dissociative disorder 1.9%, and ASD 4.8%). Of the 94 patients diagnosed with a personality disorder, 49 (52.1%) were diagnosed as personality disorder NAO, 16 (17%) borderline, 16 (17%) avoidant, 2 (2.1%) dependent, and 1 (1.1%) antisocial.

### Measures

#### C-SRS

The C-SRS (Miller and Duncan, 2004; Duncan et al., 2006) is a four-item visual analog instrument. The version for adolescents differs from the adult version of the SRS in that it uses emoticons: a smiley (positive) and a frowny face (negative). For this study, the authorized Dutch version of the C-SRS (Hafkenscheid et al., 2006) was used. The Dutch C-SRS has previously been used in research (Boon et al., 2012; De Haan et al., 2014b), and its reliability (internal consistency) was found to be satisfactory (Cronbach’s α = 0.86) (Hafkenscheid et al., 2010). As previously mentioned, therapeutic relationship was defined by three interacting elements: (1) a relational bond between the therapists, the group members, and the patient; (2) agreement on the goals of therapy; and (3) agreement on the tasks of therapy. In the C-SRS, these theoretical ideas are represented by four 10 cm visual analog scales with emoticons. Respondents are instructed to place a hash mark on a line. Negative responses are placed on the left (frowny faces) and positive responses on the right (smileys). The first item is a relationship scale, rating the session on a continuum from “The therapists and group members did not listen to me” to “The therapists and group members listened to me.” The second item is a goals-and-topics scale, rating the session on a continuum from “We did not do or talk about the things I wanted to work on or talk about” to “We did do or talk about what I wanted to work on or talk about.” The third item is an approach or method scale asking the patient to rate the session on a continuum from “I did not like the way the therapists and group members approached my problems today” to “I liked the way the therapists and the group members approached my problems today.” The fourth item examines how the patient perceived the session in total and the group alliance along the lines of “Overall, today’s session was not right for me—I did not feel part of the group” to “Overall, today’s session was right for me—I did feel part of the group.” Because the scores on the four items (the 10 cm line represents scores between 0 and 10) are added, the session total score will vary between 0 and 40: high average total scores are an indication of a high-quality therapeutic relationship.

#### Termination Status: Dropout and Completion of Therapy

In case premature termination was suggested by a patient, the patient’s family, or the treatment staff, a supportive reassessment of treatment was organized. Only when both the therapist and the patient (and family) agreed that therapy goals had been reached or when both agreed to terminate while therapy goals had only partly been reached was the patient classified as a “completer.” If the patient, therapist, or both stated that therapy was not yet completed, the exact reasons for termination were examined. In these cases, the patient was classified as a “dropout.” The intention was to classify the patients as “unilaterally terminated by the therapist” when the therapist wished to terminate therapy while the patient wished to continue. Among the included 105 patients, there were no cases of “unilaterally terminated by the therapist.” In the end, 36 patients were classified as dropouts, and 69 patients were classified as completers by the treatment staff.

### Procedure

The treatment protocol contained a programme of MBT treatments 5 days/week, with the standard weekly programme including group therapy. The C-SRS was presented to the patients at the end of each weekly group therapy session, after which it was collected and viewed by the therapist. Our purpose was to let the patients fill in the form during every therapy session. Although therapists sometimes forgot to hand out the C-SRS, in general, the C-SRS was completed during most of the group therapy sessions. The first C-SRS was completed after the first therapy session. The C-SRS that was completed during the last session (planned in the case of completers and unplanned in the case of dropouts) was marked as the last C-SRS. The total amount of C-SRS forms completed by the patient largely depended on the length of therapy.

### Statistical Analyses

All analyses were performed using SPSS, version 25.0 (IBM, 2017). First, whether the scores of the C-SRS violated the normality assumption was tested. This was not the case (skewness: dropouts.747, SE.393, *Z* = 1.90 and completers -0.315, SE.289, *Z* = 1.09; kurtosis: dropouts -0.806, SE.768, *Z* = 1.055 and completers.255, SE.570, *Z* = 0.45). Because the total scores were determined equally by all items, it was decided to exclude the item scores from further analyses.

Second, through a *t*-test, dropouts and completers were compared based on their C-SRS scores of the first session and the last session. To see if dropout was related to C-SRS scores over time, a mixed model analysis was performed with the C-SRS score as a dependent variable and time and dropout as independent variables.

Third, the reliable change index (RCI) for the C-SRS was calculated using the Jacobson and Truax (1991) formula. The standard error of measurement (SEM) was calculated (*s*√1 - *rxx*), DIFF = √2(SEM2), RCI = xt1 - xt2/SDIFF. A 95% reliability interval was used. The resulting categories were “deteriorated” (a significant decrease in score between two sessions), “no reliable change” (no significant increase or decrease in score between two sessions), and “improved” (a significant increase in score between two sessions). Based on all questionnaires (*N* = 2,378) with a reliability (Cronbach’s alpha) of.921 and *SD* of 8.15, the standard error was 3.24. The reliable change criterion was (1.96 ^∗^ 3.24) 6.35.

Fourth, a generalized estimating equation (GEE) analysis with an exchangeable working correlation matrix was performed to see if a decrease in C-SRS score could predict dropout, with the dichotomous variable “significant decrease (RCI) in the C-SRS score between two consecutive sessions” as the independent variable and “dropout within three sessions” as the dependent variable.

Last, a forward binary logistic regression analysis was performed with dropout vs. completer as the dependent variable and gender, age, diagnoses, and significant decreases (using RCI) during the last five sessions of therapy as independent variables.

## Results

### Descriptives

The 105 subjects attended group psychotherapy between March 2013 and October 2018, with an average number of group members of 5.0. The number of C-SRSs completed per participant ranged from 2 to 43 times (*M* = 22.07, *SD* = 10.45). The number of missed sessions (*M* = 3.53, *SD* = 4.97) was calculated by subtracting the attended sessions (*M* = 25.6, *SD* = 12.7) from number of planned sessions (*M* = 26.3, *SD* = 12.7). Based on the results, the percentage of missed sessions was calculated for each respondent. This percentage did not differ (*p* = 0.72) between completers (2.78%, *SD* = 0.58) and dropouts (3.27%, *SD* = 1.51). Of the 2,832 attended sessions, 2,367 C-SRS were completed (response 83.6%).

### Dropouts vs. Completers

The treatment duration of the dropouts (*M* = 125.56 days, *SD* = 99.1) was significantly (*t* = 7.497, *p* < 0.001) lower than that of the completers (*M* = 261.91 days, *SD* = 63.3). Dropouts (*N* = 36) completed the C-SRS on average 13.42 times (*SD* = 11.38), and completers (*N* = 69) completed it on average 26.58 (*SD* = 6.33) times. These numbers differed significantly (*t* = 7.629, *p* < 0.001). Table 1 presents a comparison between first- and last-session scores of the C-SRS completers and dropouts. For completers, the C-SRS scores increased significantly between the first and last sessions, while the scores of the dropouts did not change. All the individual items of the C-SRS contributed equally to these results: a significant increase for completers and no significant change for the dropouts.

**TABLE 1:** Comparison first- and last session scores C-SRS completers and dropouts.

	***N***	**SRS 1st session**		**SRS last session**			
		
		***M***	***SD***	***M***	***SD***	***t***	***p***
Completers	69	27.30	6.67	32.34	6.41	4.84	0.001
Dropout	36	26.47	7.45	23.83	9.69	1.44	0.159
Total	105	27.01	6.92	29.42	8.66		

No significant difference was found (*t* = 0.583, *p* = 0.577) on the first C-SRS scores for dropouts vs. completers. The scores of the last session however differed significantly (*t* = 4.756; *p* < 0.001, Cohen’s *d* = 1.035) between both groups. Total C-SRS scores decreased by 0.86 points per session on average for the dropouts, while increasing by 0.18 points per session for the completers.

Mixed model analyses showed no differences (*p* = 0.665) in C-SRS scores over time between dropouts and completers, implying that dropout cannot be predicted from the progression of C-SRS scores. A GEE analysis did not reveal dropout to be a significant predictor of significant (RCI) decreases in C-SRS scores (*p* = 0.730). The next step was to identify the last five sessions of therapy and compare the differences in C-SRS scores between these sessions. No differences were found between completers and dropout in comparison of the fifth- and fourth-last sessions. On the other hand, comparison of the third-last and the second-last sessions showed that 7.1% (*n* = 3) of the completers (*N* = 42) had a significant (RCI) decrease in C-SRS score between these sessions, while for dropouts (*N* = 17), this was 35.3% (*n* = 6) (*df* = 1, χ^2^ = 7.419, *p* = 0.006). Subsequently, a comparison of the C-SRS score of the second-last and the last sessions showed that 4.0% (*n* = 2) of the completers (*N* = 50) had a significant (RCI) decrease in C-SRS score between these sessions, while for dropouts (*N* = 30), this was 26.7% (*n* = 8) (*df* = 1, χ^2^ = 8.808, *p* = 0.003) (Table 2). In conclusion, during the last three sessions, 7.2% (*n* = 5) of the completers showed a significant decrease in C-SRS scores, compared to 38.9% (*n* = 14) of the dropouts (*df* = 1, χ^2^ = 15.98, *p* < 0.001). Binary regression analysis (forward stepwise) shows that the model that fitted the data (χ^2^ = 5.50; *p* = 0.019) included only a significant decrease between the second to last and the last sections (*p* = 0.035) with an odds ratio (Exp B) of 4.38.

**TABLE 2:** RCI between first- and last session scores C-SRS for completers and dropouts.

	**Completers**		**Dropouts**		**Total**	
	***N***	**%**	***N***	**%**	***N***	**%**
Significantly increased	28	40.6	9	25.0	37	35.2
No significant change	36	52.2	12	33.3	48	45.7
Significantly decreased	5	7.2	15	41.7	20	19.0

The differences between sessions for all respondents for all sessions (*N* = 1906) showed that 324 times (14.3%) a significant decrease (RCI) in C-SRS scores occurred. Table 2 shows the significant (RCI) decreases between first- and last-session C-SRS scores for completers and dropouts.

## Discussion

The aim of this study was to gain deeper insights into the association between the quality of the therapeutic relationship and treatment termination status among high-risk adolescents receiving intensive MBT. We measured the therapeutic relationship during group therapy with the C-SRS, through which the adolescent rated the therapeutic group alliance. No differences were found in the initial scores of the C-SRS, indicating that dropouts and completers did not differ in the way that they experienced the therapeutic alliance at the start of therapy. On the other hand, the development of C-SRS scores during the course of therapy did differ for the two groups: completers showed improving scores of the therapeutic relationship during the course of therapy, while dropouts showed declining scores. These differences occurred mainly at the end of the treatment course. The results indicate that an improving therapeutic relationship during the course of therapy is associated with adherence to therapy, while a decreasing quality of the therapeutic relationship during the course of therapy is associated with the patient ending therapy prematurely (Norcross and Lambert, 2018). Furthermore, a significant drop in the therapeutic relationship between the next to last and final sessions was four times more likely to occur. This could mean that even though clients are about to drop out, they will still attend another session, giving the therapist a chance to repair the rupture in the therapeutic relationship. Our study showed that the rather short instrument (C-SRS), which can be easily applied in clinical practice to be completed by adolescent patients themselves, is a valuable instrument for measuring the quality of the therapeutic relationship.

A significant decrease in the therapeutic alliance in the last three sessions was a predictor of dropout. For dropouts, such a decrease occurred in 38.9% of the cases, while for completers, this was 7.2% of the cases. Because such a significant decrease in therapeutic group alliance occurred during the treatment process in 14.3% of all cases, only with hindsight was it clear that such decrease has led to dropout. To prevent dropping out of therapy, this means that every substantial decrease in C-SRS score is worth discussing. In this study, some participants spoke of being satisfied with the session, while on the C-SRS, they rated the therapeutic alliance of that same session as low. Probably, these patients avoided being openly honest about their negative feelings toward the group and therapist. By using the C-SRS, such unspoken inconsistency can be recognized, understood, and worked through in the next session, and thereby outcomes can be improved (Norcross and Lambert, 2018). In case the decrease in C-SRS score is caused by something that occurred in the working alliance with the therapists and/or the group members, differences in perspective and thoughts, beliefs, wishes, and feelings can be explored and validated (Bateman and Fonagy, 2012). This way, group psychotherapy is a shared attentional process which strengthens mentalizing capacities and interpersonal functioning.

We found that dropouts and completers did not differ in the way they experience the therapeutic alliance at the start of therapy, which is not in line with most research on this topic (De Haan et al., 2013). This could be explained by the emphasis placed on internal motivation prior to treatment. Among others, adolescents in this study were asked during the registration phase to write a motivation letter for the treatment. For youth therapists, paying attention to therapeutic alliance in general and especially at the start of the treatment may be particularly relevant due to distrust of adult authorities and a desire for autonomy (De Haan et al., 2013). Limitations of this study must be mentioned. The first limitation is that the generalizability of the results found in a sample of high-risk adolescents in group psychotherapy with other patients with personality pathology and patients with other pathology is yet to be determined. Second, it is not clear if the dropouts are the result of a decrease in the therapeutic alliance or a result of other factors, potentially even outside of the treatment. Third, the fact that in some cases one of the group therapists was also an attending patient’s individual therapist could influence the results. Fourth, the significant differences in treatment duration in the relatively small sample could influence the results due to diversity. Nevertheless, the C-SRS can help psychotherapists to timely intervene when breaks, which may lead to dropout, occur in the therapeutic alliance with adolescents with personality pathology. Future research on all elements of the therapeutic alliance including the view of the therapist for high-risk adolescents is needed.

## Conclusion

In this study, the association between the quality of the therapeutic relationship from the patients’ point of view and dropout was examined in a seldom-studied adolescent group with personality pathology. This high-risk group of patients is often excluded from scientific research, although personality disorders often start and peak in middle to late adolescence (Hutsebaut et al., 2013; Shiner and Allen, 2013). The psychosocial and economic burden is high (Feenstra et al., 2012; Chanen and McCutcheon, 2013). Against this background, clinical practice is in need of more information regarding this difficult group. It is to be expected that the number of breaks in the therapeutic alliance are higher in this group than in a group with less severe psychopathology (Eubanks and Muran, 2018). Furthermore, as the size of the therapist effect—one element of the therapeutic alliance—is strongly related to initial client severity (Norcross and Lambert, 2018), it is also to be expected that the therapist effect in this adolescent group is substantial.

## Data Availability Statement

The datasets generated for this study are available on request to the corresponding author.

## Ethics Statement

Ethical review and approval was not required for the study on human participants in accordance with the local legislation and institutional requirements. Written informed consent to participate in this study was provided by the participants’ legal guardian/next of kin.

## Author Contributions

KH performed the data collection and wrote the manuscript. AB contributed to the design of the research project, performed the statistical analyses in the study, and revised the manuscript. RV oversaw the research project and reviewed the manuscript. All authors read and approved the final manuscript.

## Conflict of Interest

The authors declare that the research was conducted in the absence of any commercial or financial relationships that could be construed as a potential conflict of interest.
